# Recent Outbreak of Monkeypox: Implications for Public Health Recommendations and Crisis Management in India

**DOI:** 10.7759/cureus.45671

**Published:** 2023-09-21

**Authors:** Astha Kumar, Sonali K Borkar, Sonali G Choudhari, Harshal G Mendhe, Nandkishor J Bankar

**Affiliations:** 1 Community Medicine, Datta Meghe Medical College, Nagpur, IND; 2 Community Medicine, Jawaharlal Nehru Medical College, Wardha, IND; 3 Microbiology, Jawarhal Nehru Medical College, Wardha, IND

**Keywords:** vaccines, pandemic, smallpox, outbreak, zoonosis, monkeypox

## Abstract

Monkeypox is a rare and self-limiting disease that was eradicated globally through vaccination approximately forty years ago, following the eradication of smallpox. The purpose of this article is to explore the implications of the recent monkeypox outbreak on public health recommendations and crisis management in India. An overview of the consequences of the current monkeypox epidemic on public health, epidemiology, clinical findings, management, challenges, and existing strategies for this disease, along with recommendations are discussed. It is crucial to develop evidence-based recommendations for the diagnosis and treatment of monkeypox, as well as early case identification and contact tracing. To prevent the spread of infection, travelers from affected countries should be subjected to health testing and quarantine. In order to successfully control the outbreak, a multidisciplinary team should be established to manage the monkeypox virus at tertiary care facilities, and health workers with occupational exposure to the virus should be assessed and given management plans.

## Introduction and background

In 1958, this virus was first isolated in a laboratory from monkeys, so the virus was known as the monkeypox virus [1]. An orthopox viral family includes this virus [2]. This virus resembles the variola virus in some of its features. Currently, two different strains of this virus have been identified, the first is the West African strain, which is very mild and has a lower fatality rate, and the second one is the Congo basin strain, which has a higher fatality rate. In 1970, the Congolese Republic announced the first-ever human infection case. In recent years, since March 2023, about 113 countries reported 93516 cases of the Monkeypox virus [3]. Therefore, to prevent its further spread in the community, it is necessary to develop a system that is capable of facilitating a rapid and accurate diagnosis of this disease and knows who to call to report these cases and how to advise the patient along with providing proper treatment for the disease [1]. This article outlines the importance of public health, epidemiology, clinical findings, management, challenges, and existing strategies for this monkeypox virus along with a few recommendations.

Public health significance of monkeypox 

Given the thought that monkeypox is a rare and self-limiting sickness, little has been done to cater to the disease ever since its identification more than seven decades ago [4]. Although an increasing public health issue associated with monkeypox has indeed been detected, notably in West Africa along with the places where proximity is evident contact between humans and wildlife reservoirs and in particular which seem to be signs that perhaps the frequency of illness attacks is increasing [5]. In recent years, both the frequency and trend of cases significantly increased. Regarding the onset of symptoms, the timeframe of the diagnostic and therapeutic profile is analogous to the symptoms of smallpox in terms of the appearance and spread of rashes. However, monkeypox is typically less serious than smallpox compared to complications, case fatalities, & scarification levels. Several reports have raised concerns regarding the appearance of this virus in addition to how similar its clinical presentation is compared to smallpox, a fatal illness which has been eradicated worldwide through vaccination forty years ago after the global eradication of smallpox [6]. This monkeypox virus might be prevented by the smallpox vaccination, it’s cases increased when the delivery of the smallpox vaccination was stopped. All through outbreaks has indeed proven to be problematic to distinguish this virus from chickenpox and independent herpesvirus therapeutically. Nonetheless, occasional zoonotic viruses along with several additional orthopox viruses are likewise concerning, Buffalopox epidemics with several human instances have been documented across India [7]. The aim of the review is to identify what are the implications of the recent outbreak of monkeypox for public health recommendations and crisis management in India.

Methodology

**Figure 1:**
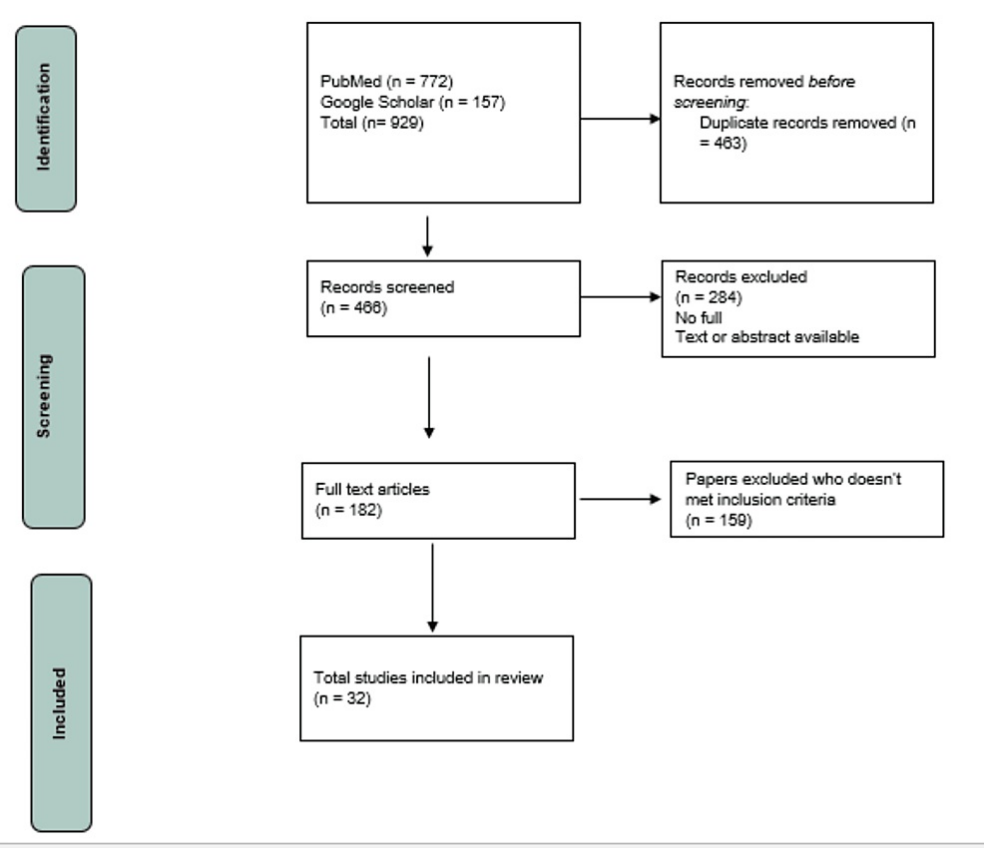
PRISMA flow chart

The relevant databases for this study include PubMed, and Google Scholar along with the websites of the World Health Organization, Centre for Disease Control, National Centre for Disease Control, Integrated Disease Surveillance Program, and Integrated Health Information Portal. The search terms were developed based on the research question and will include variations of "monkeypox," "outbreak," "India," "public health," and "crisis management." The search was carried out in the identified databases using the search terms. To guarantee relevance to the ongoing outbreak, the search was restricted to research published within the previous ten years. Additionally, only articles in English were included in the search. On the basis of pre-established criteria, the studies were evaluated for inclusion or exclusion. The inclusion criteria were to include studies that examine the recent outbreak of monkeypox, its implications for public health recommendations and crisis management, and provide recommendations for future outbreaks. Exclusion criteria were to exclude studies that aren't significant to the research question, studies that are not in English, and studies that have not undergone peer assessment. Full-text publications were obtained for all studies that met the inclusion criteria were obtained. A total of 41 articles were found amongst which 21 were case reports and 20 were narrative reviews out of which final studies reviewed for this article were 32 as shown in Figure 1 PRISMA. Data were extracted from full-text articles. The information contains research attributes (such as the researcher, and study design used along with the year of publication), as well as participant attributes (such as sample size and demographics), intervention/exposure, outcome measures, and major results. The findings from the included studies were synthesized to form an overall understanding of the consequences of the current monkeypox epidemic for public health recommendations and crisis management in India. 

## Review

Epidemiology

The clinical manifestation of this virus amongst humans was first discovered in the year 1970 in Basankusu, in the Democratic Republic of the Congo [5]. The earliest episode of this viral disease was discovered in 1958, in an Asiatic Macaca fascicularis which was transported from Malaysia to a laboratory in Copenhagen city of Denmark for polio vaccine testing two clades of this virus in West Africa and the Congo Basin were explored [8]. In contrast to the West African species, which possesses a reduced rate of case fatalities of about 0.3%, the Congo Basin clade generates more serious illnesses and has a greater case fatality rate of 11% [9,10]. African nations such as Ghana, Liberia, Benin, Nigeria, etc. have a high prevalence of this viral disease. It's unclear how serious this disease has affected public health. Following the smallpox eradication a five-year tenure in the year 1980 intensive surveillance in the Congo revealed three hundred thirty-eight cases. From 1970 to 1980, fifty-nine cases were documented. In 2003, imported Ghanaian Gambian giant rats contaminated field dogs in the Midwest of the U.S. resulting in 53 human infections as the first instance from outside Africa [11]. The 2005 breakout in Sudan is the second instance of monkeypox that occurred without reference to the West African and Congo Basin areas [12]. A hundred and twenty-two cases were reported in Nigeria in 2017 [13]. Global outbreaks are frequently traced to travelers who just got back from endemic regions. A fresh epidemic was discovered by the World Health Organization (WHO) in May 2022. By June 2, 2022, there were above 800 laboratory-confirmed incidents from twenty-seven nations that were not even endemic for the illness and didn't have a background of mobility to endemic regions [3]. Centre for Disease Control (CDC) reported 5,783 cases that have been verified for this virus since July 1, 2022, which spread across fifty-two different nations. The natural host of the monkeypox virus is uncertain, the reservoir of this virus is yet to be studied more, but it is clear that this virus circulates in nature.

Modes of transmission

Several modes this virus is believed to propagate, and they all entail direct contact with sick people or wildlife carrying this virus [14]. Being exposed to contaminated animals' feces may constitute a major contributor to the risk of developing this disease [15]. Spread from an animal to a person can happen through interacting directly or engagement involving infected animals. All of that is typically brought on by biological fluids such as nasal secretions, saliva, or pus [16] Among humans, the disease is primarily spread by coughing or sneezing, sustained one-to-one exposure, or coming into touch with infected lesions [17]. Some contaminated materials or products that are considered to enhance the likelihood of fomite-borne transmission are high among residents of a similar property like sharing a dwelling or even using crockery that was previously utilized by an infected patient. Homosexuals have a higher probability of contracting this viral disease [18]. The World Health Organization states that although it is unknown whether monkey pox spreads through sexual contact or not, close contact is a major factor in transmission [19]. The incubation period for this monkey pox disease is six to thirteen days, lesions usually appear at the skin and oropharynx region and its virus transports throughout the body via the bloodstream while the site of inoculation of the virus in the body acts as a reservoir for this disease [17].

Clinical findings

Monkeypox disease presents with various systemic as well as generalized symptoms as depicted in Table 1, such as nausea, vomiting, fever, Hepatomegaly, septicemia, dehydration, breathing difficulties, respiratory infections, disorientation, rashes, severe cervical lymphadenopathy, dysphagia, ocular discomfort, optical asymmetry [20,12]. The prodromal and the rash are the two distinct stages of monkeypox in humans. Infection with the monkeypox virus causes the following initial symptoms: headache, fatigue, temperature, shivering or sweating, throat infection, muscle aches, and lymphadenopathy the dermal manifestation often emerges just several days following lymphadenopathy and a temperature [21]. A rash is characterized by blisters that customarily begin from the face following proliferation to the entire body [22].

**Table 1:** Symptomatic phases of monkeypox

Invasion phase	Skin eruption phase
Duration = 0-5 days	Duration = 1-3 days
Febrile stage ( high fever)	Rashes on the face, palm, sole, oral mucosa, ano genital areas
Intense headache	Rashes may be a few to thousands in numbers
Lymphadenopathy (exclusive)	The rash starts as a macule & converts into a papule
Myalgia	A vesicle is formed which gets filled up with pus to form a pustule
Back pain	Crusting occurs which ultimately dries and falls off

Differential diagnosis

Table 2 shows that three viral infections-monkey pox, chickenpox, and measles-can all emerge as skin symptoms, but they each have unique characteristics that help with differential diagnosis. Systemic symptoms, a history of animal contact, and a "stair-step" pattern of lesions on the face, trunk, and limbs are all characteristics of monkeypox. Concentrated lesions on the trunk, severe itching, and minor respiratory problems are common signs of chickenpox. The symptoms of measles include a rash that begins on the face, spots called Kopliks in the mouth, systemic symptoms, and a typical pattern of spreading down the body. It is possible to distinguish between these three illnesses using distinct skin lesions, distribution, accompanying symptoms, and exposure history, which enables precise diagnosis and therapy [4].

**Table 2:** Differential diagnosis

Clinical presentation	Monkeypox	Chickenpox	Measles
Fever	1 to 3 days prior to the appearance of rash	1 to 2 days prior to the appearance of rash	3 to 5 days prior to the appearance of rash
Leison	Singular phase	Numerous phase	Numerous phase
Dermal signs	Appears late	Appears early	Appears early
Rash distribution	Much denser in the face, palm, and soles	Denser on the trunk, not present in the palm and soles	Starts from the face and then moves to the extremities
Lymphadenopathy	Present	Absent	Rare
Death	10%	Rare	Variable

Treatment and management

The foundation of therapeutic treatment for this disease is supportive & symptomatic treatment [22]. There are presently no particular treatments for this illness, however, the vaccinia immunoglobulin, cidofovir, vaccinia vaccine, and tecovirimant may be useful for managing the condition and has been approved by the European Medicines Agency for the management of monkeypox virus in 2022 [23,24]. Vaccine immune globulin is a clear fluid that is made from highly concentrated IgG antibodies antagonistic towards the vaccinia virus that was recovered from physically fit individuals who have earlier undergone a vaccination for the live vaccinia virus [25]. To prevent the infection, the CDC has given the go-ahead to employ viral immunoglobin. The CDC's Emergency Access Investigational Novel Program permits the consumption of tecovirimat for illnesses brought on by monkeypox [10]. The guideline also authorizes opening an ingestible capsule and merging its ingredients with any fluid or mushy foodstuff for young kids measuring below 13 kg. Both an injectable vial and an oral-capsule version of tecovirimat with a dosage of 600 mg twice a day (BD) advised intake for two weeks are available from the Strategic National Stockpile [10,26]. The Orthopoxvirus protein p37 is prevented from generating viral replication by tecovirimat, limiting the virus' ability to propagate within a system of the host [27]. The majority of monkeypox management focuses on treating symptoms, avoiding complications, and preventing the reoccurrence of infection. Mild presumed or verified cases should be segregated and treated symptomatically and supportively as necessary [20]. Adequate infection control measures must be enacted mainstay of treatment is sufficient diet, fluids, and antipyretics. Continuous monitoring of skin eruptions must be done for bacterial infections which are secondary [28]. Children and females, who pose a higher risk of infection, must be taken to a healthcare facility for more frequent observation and medical attention [29].

Challenges and importance of global surveillance and monitoring for monkeypox outbreaks

Worldwide Information and monitoring platforms for surveillance of this monkeypox outbreak have encountered several difficulties over the years, including cases without symptoms that go undetected, poorly skilled personnel, poor research lab assistance of contagious illnesses in nations where this viral disease was announced endemic, misreporting because of insufficient healthcare services in developing nations, an absence of global cooperation, with nations' unwillingness to offer comprehensive data because of the political as well as financial repercussion [15]. To correctly evaluate the public health consequences and establish policies to lessen the likelihood of transmission of the virus, emphasis is generally given to advanced surveillance platforms, demographic assessment, and universal distribution of therapeutics and immunizations particularly in middle- and lower-income regions [30]. 

Existing management strategies for monkey pox outbreak in India

The Ministry of Health and Family Welfare (MoHFW) issued a protocol for monkeypox disease. Diligent surveillance and case detection are strongly emphasized in these guidelines. India has designated the National Institute of Virology located in Pune as the center for investigating the monkeypox virus. WHO has also published an emergency management guide for handling outbreaks of monkeypox. Furthermore, the National Centre for Disease Control (NCDC) has made suggestions for limiting the monkeypox outbreak in India. The nation's monkeypox virus epidemic is regularly tracked by the Integrated Disease Surveillance Program (IDSP), which is displayed on the Integrated Health Information portal [31]. In three major government hospitals in Delhi-Vardhman Mahavir Medical College (VMMC) Safdarjung, Ram Manohar Lohia (RML) Hospital, and Lady Hardinge Medical College-the government has also made steps to make isolation rooms operational [32]. The number of healthcare facilities has now increased to three: Dr. Baba Saheb Ambedkar Hospital, Lok Nayak Jai Prakash Narayan Hospital, and Guru Teg Bahadur Hospital The Indian Council of Medical Research (ICMR)-Department of Health Research authorized 15 more virology institutes and laboratories to undertake confirmatory testing for the monkeypox virus, with AIIMS Delhi virology lab serving as the nodal center for verifying the cases by reverse transcription-polymerase chain reaction (RT-PCR). Many well-known institutions include the National Institute of Cholera and Enteric Diseases in Kolkata, Government Medical College (GMC) Thiruvananthapuram, Kasturba Hospital for Infectious Diseases in Bombay, and the National Institute of Virology (NIV) field unit in Kerala [28].

Implementing the one health strategy to combat the spread of new and re-emerging zoonotic diseases

By adopting one health strategy of WHO diseases monkeypox can be tackled [33]. The fast spread of transmissible infections is a result of the expanding global population and greater international travel [34]. Through improving awareness initiatives, stigmatization related to ignorance can be avoided. High-risk persons should receive vaccines, and efforts to identify new drugs should be stepped up to fight such diseases [35]. The existing small poxvirus vaccines can be quite helpful in prophylaxis, but they must be administered with caution in individuals with immunological deficiencies. It will be vital to stop the spread of this disease using alternatives such as vaccinia immune globulins, herbal traditional remedies, and novel discoveries. Globally research organizations and the pharmaceutical industry should devote greater resources to the study of novel antiviral agents that target various viral life cycles [36]. Live, replication-incompetent vaccinia virus and Live, replication-competent vaccinia virus are the two vaccines that are now available. However, most cases of the virus just require supportive care. Within four days of the patient's initial exposure, the WHO advises post-exposure prophylaxis (PEP) with a vaccine given to the patient's contacts [37,38].

Treatment options include antiviral medications cidofovir, tecovirimat, and bricidofovir along with VIGIV (vaccinia immune globulin intravenous) [39].

Predicting and preventing monkeypox outbreaks: approaches and challenges

Predicting future outbreaks of monkeypox can be a challenging task, as it depends on a variety of factors such as the prevalence of the virus in animal populations, the level of human contact with infected animals, and the effectiveness of public health measures to contain and control the spread of the disease. However, several approaches can be used to monitor and detect the emergence of monkeypox outbreaks, including:

Surveillance of Animal Populations

As a zoonotic disease, monkeypox generally spreads from animals to humans. Keeping track of the virus' frequency in animal groups, especially those of rodents and primates, can help predict outbreaks before they happen [40]. 

Monitoring Human Cases

Monkeypox cases in humans can be monitored to learn more about the disease's spatial and temporal trends. Public health professionals can utilize this measure to recognize outbreak hotspots and to initiate appropriate preventative measures [40].

Environmental Surveillance

It includes testing for the monkeypox virus in soil, water, and other environmental sources. Despite the fact that no cases of the virus in humans or animals have been recorded, this method can still be used to identify locations where the virus can reside in the wild [41].

Early Detection and Rapid Response

In order to restrict epidemics from spreading, early diagnostic systems and response procedures should be established. As part of this, healthcare providers are trained to identify the signs and symptoms of the virus. Fast action strategies such as isolation of patients, contact tracing, and vaccination are implemented [3].

Collaboration and Information Sharing

Teamwork involving public health authorities, veterinary services, and other stakeholders can aid monitoring and response operations. Early diagnosis and reaction to monkeypox outbreaks can be facilitated by exchanging data on cases and the virus's genetic makeup [3].

Overall, predicting future monkeypox outbreaks requires a multi-faceted approach that involves surveillance, monitoring, and collaboration between various stakeholders [19]. By implementing these strategies, public health authorities can help prevent and control the spread of monkeypox, protecting both human and animal populations.

## Conclusions

In conclusion, addressing the current monkey pox outbreak in India requires a comprehensive and coordinated approach that involves strengthening the healthcare system, increasing awareness and training of healthcare workers, conducting genomic surveillance, establishing diagnostic laboratories, and building institutionalized isolation shelters it is also crucial to develop recommendations for diagnosis and treatment based on evidence, early case identification, and contact tracing. Healthcare professionals must be provided with personal protective equipment to prevent transmission and handle samples with care. Vaccination should be made available to those most at risk, and routine immunization of children is recommended. Travelers from affected countries should be subjected to health testing and quarantine to stop the infection from spreading. To successfully control the outbreak, a multidisciplinary team for managing the virus at tertiary care facilities should be established, and health workers with occupational exposure to the virus should be assessed and given management plans. The misdiagnosis of disease as other skin conditions must also be addressed. By implementing these measures, it is feasible to halt and limit the spread and protect public health in India.
